# Onion-Structured Si Anode Constructed with Coating by Li_4_Ti_5_O_12_ and Cyclized-Polyacrylonitrile for Lithium-Ion Batteries

**DOI:** 10.3390/nano10101995

**Published:** 2020-10-10

**Authors:** Arailym Nurpeissova, Aliya Mukanova, Gulnur Kalimuldina, Nurzhan Umirov, Sung-Soo Kim, Zhumabay Bakenov

**Affiliations:** 1 National Laboratory Astana, Nazarbayev University, Nur-Sultan 010000, Kazakhstan; aliya.mukanova@nu.edu.kz; 2 School of Engineering and Digital Sciences, Nazarbayev University, Nur-Sultan 010000, Kazakhstan; gkalimuldina@nu.edu.kz; 3 Graduate School of Energy Science and Technology, Chungnam National University, 99 Daehak ave., Yuseong-gu, Daejeon 34134, Korea; nurzhan.umirov@nu.edu.kz (N.U.); kimss@cnu.ac.kr (S.-S.K.)

**Keywords:** silicon nanoparticles, LTO, cPAN, onion-like structure, nanostructures, energy storage

## Abstract

Low dimensional Si-based materials are very promising anode candidates for the next-generation lithium-ion batteries. However, to satisfy the ever-increasing demand in more powerful energy storage devices, electrodes based on Si materials should display high-power accompanied with low volume change upon operation. Thus far, there were no reports on the Si-based materials which satisfy the stated requirements. Hence, here, we report on modified onion-structured Si nanoparticles (SiNPs) co-coated with Li_4_Ti_5_O_12_ (LTO) and cyclized polyacrylonitrile (cPAN) to bring the synergistic effect enhancing the conductivity, tolerance to volume change and stable performance. Obtained results suggest that the nanoparticles were conformally coated with both materials simultaneously and the thicknesses of the films were in a range of a few nanometers. Electrochemical tests show that the modified SiNPs deliver a high initial capacity of 2443 mAh g^−1^ and stable capacity retention over 50 cycles with 95% Coulombic efficiency.

## 1. Introduction

Batteries are the most commonly used devices for powering portable consumer electronics, electric vehicles and large-sized stationary energy storage systems [1]. Compared to other traditional rechargeable batteries, lithium-ion batteries (LIBs) are favored due to their high energy density, long service life, low-weight and impeccable compatibility with the environment. Nevertheless, it is not a secret that currently available LIB technology is already incapable of keeping up with the progressive innovative technologies that require a way more higher power capabilities than the LIBs can provide. Ways to circumvent this problem might lay in the exploration of novel materials, trial of beyond lithium chemistries or enhancement of existing battery components. The latter being the most researched, thousands of works on the improvements of battery components separately or collectively were reported.

Silicon (Si), in the role of a promising candidate for anode materials, is being investigated continuously owing to advantages as high theoretical capacity (3579 mAh g^−1^), low potential, environmental compatibility, low level of toxicity and reduced cost [2,3,4]. Yet, practical implementation of this material in LIBs is restrained by several critical issues stemming from the intrinsic properties, including poor electronic-ionic conductivity and huge volume changes which leads to the destruction of microstructure during cycling [5,6]. The stated problems can be diminished or eliminated by reducing the particle size to a nanometer range, surface modifications or corresponding structural adjustments [7,8,9].

Diverse nanosized Si materials have been designed and fabricated, that is to say Si nanoparticles (SiNPs) [10], Si nanowires (SiNWs) [11], Si nanotubes (SiNTs) [12], Si thin films (SiTFs) [13,14], Si hollow structures [15,16,17] and so forth. Nanosized structures offer tolerance to volume change, high surface area and improved electrode kinetics. Nevertheless, the fracture during continuous cycling cannot be totally avoided even in low-dimensional Si materials. Consequently, modifications achieved by coupling the SiNPs with carbon coating [17,18], metals, metal oxides [19,20,21], polymers [22,23] and other materials were described. Exhibiting distinctive physico-electrochemical characteristics, these materials can exert distinct impacts on the electrochemical performance of the SiNPs. Remarkably, combining the SiNPs with the stated above materials (active or inactive) endowed a higher electrochemical performance than the pristine SiNPs [10]. While some materials give structural integrity to the SiNPs others help to improve the conductivity. Thus, determining the divergent types and electrochemical effects of combined materials is very important in order to optimize the design and fabrication to specifically target the volume change and conductivity in Si. Also, it is worth mentioning that the combining process should not be simply mixing but rather physical or chemical methods to better incorporate the materials to form composites. Although different routes were discussed and researched to make SiNPs suitable for commercialization, there is still a long way to go. Drawbacks related to the volume change are still needed to be overcome.

To break through the limitations concerning SiNPs, Liu et al. proposed a core-shell structured Si-LTO [24] while Lee and co-workers reported on a novel Si-based multicomponent design, which consist of the Si core and multifunctional shell layers [25]. The composite materials delivered high capacity suggesting that the coating helped to improve the performance providing stable structural integrity. Another works dedicated to the coating of Si with polyacrylonitrile (PAN) rendered a very promising approach to solve the problems [26,27]. PAN is one of the widely used and easily synthesized semicrystalline polymers with strong interaction between nitrile groups. It possesses excellent properties as thermal stability, resistance to most organic solvents and found its various applications in LIB improvements.

Inspired by these works, herein, we present the preparation of onion-like structured particles where SiNPs are covered by spinel Li_4_Ti_5_O_12_ (LTO) and cyclized polyacrylonitrile (cPAN) using simple and robust liquid-phase coating. Two shells demonstrate a synergistic positive effect on the performance of SiNPs. LTO has a unique structure with almost zero deformation during lithiation and delithiation processes. cPAN has elastic polymeric properties as well as good conductivity. Thus, combination and synergetic effect of this two-shell structure provides SiNPs with the ability to withstand the volume change and enhances the conductivity.

## 2. Materials and Methods

The process of coating SiNPs involved two steps: (1) coating with LTO with the consequent (2) coating with cPAN. In a simple process pristine SiNPs (Guangzhou Jiechuang Trading Co., GuangZhou, China, 30–50 nm) were treated with hydrogen peroxide (H_2_O_2_, Sigma Aldrich, St. Louis, MO, USA) and dried in a convection oven at 70 °C. The washing was done in order to activate the SiNPs surface with a hydroxyl group so that Si can mix well with Li and Ti precursor solutions during the coating process. Then, 0.5 g of the Si powder after treatment and 0.82 g (8 mmol) of lithium acetate dihydrate (CH_3_COOLi*2H_2_O, Sigma Aldrich, St. Louis, MO, USA) were dispersed in 10 mL of ethanol and mixed using a magnetic stirrer. After 1 h, 1.4 g (5 mmol) of titanium (IV) isopropoxide (C_12_H_28_O_4_Ti, Sigma Aldrich, St. Louis, MO, USA) was added to the above solution and continuously stirred for 2 h. Then, the resulting precipitate was filtered and dried in a convection oven at 70 °C. The resulting mixture, the precursor, was then ground using an agate mortar. Finally, the powder was heat treated in a tubular furnace at 610 °C for 12 h under argon to form lithium titanate on the surface of SiNPs, that is, to obtain LTO coated SiNPs. For the subsequent synthesis of the Si-LTO-cPAN anode material, LTO coated SiNPs and PAN (J&K Scientific, San Jose, CA, USA, MW 150,000) were mixed in a weight ratio LTO-Si:PAN = 3:7 in a dimethylformamide solvent so that the weight content of all solids was equal to 87% of the total weight. The resulting viscous mixture was then stirred by a magnetic stirrer for 12 h and then placed in a tubular furnace for annealing in argon at a temperature of 300 °C for 12 h. Produced black substance was crushed and ground in an agate mortar.

The crystal structure of pure and coated SiNPs was probed with XRD using an X-ray diffractometer (XRD, SmartLab, Rigaku Co., Tokyo, Japan, Cu-Kα radiation (λ = 0.15406 nm), sweep speed 0.02 deg·min^−1^, 2θ range between 20–80°). Morphology of the powders was investigated with scanning electron microscopy (FE-SEM, JEOL JSM-7500F, Tokyo, Japan). Si-LTO-cPAN powder was studied using energy dispersive X-ray spectroscopy (TEM-EDX) coupled to transmission electron microscopy (TEM). To confirm the cyclization of PAN, Raman spectroscopy (LabRAM HR Evolution, HORIBA, Tokyo, Japan) was utilized.

To prepare a working electrode, a suspension consisting of 80% by weight of a composite Si-LTO-cPAN, 10% of Ketjen Black (KB) and 10% of carboxymethyl cellulose (CMC, MTI) dispersed in deionized water was mixed in a planetary vacuum mixer (ARV-310 CE, Thinky corporation, Tokyo, Japan) at 1200 rpm for 10 min. Then, the resulting mixture was applied to Cu foil using the Doctor Blade (50 μm) spreading technique and dried in a vacuum oven for 2 h at 110 °C. To assemble coin-cells, disks with a diameter of 16 mm were cut from dried electrodes. The active material masses were calculated to be approximately 0.5 mg. A 1 M solution of lithium hexafluorophosphate (LiPF_6_) salt in a mixture of ethylene carbonate/ethyl methyl carbonate/dimethyl carbonate (EC/EMC/DC) in a volume ratio of 1:1:1 (SoulBrain MI, Northville, MI, USA) was used as an electrolyte and a Celgard-2300 multilayer propylene separator was used as a separator. Lithium (Li) metal acted as both a reference and counter electrodes. The three components, the prepared anode, separator and Li metal were superimposed on each other with the addition of electrolyte in the CR2032 type coin-cell. The whole process was carried out in the argon-filled glove box (MasterLab, MBraun, Garching, Germany) since humidity and air can react with materials and lead to their oxidation and/or degradation.

To test the electrochemical activity of the assembled coin-cells, methods such as cyclic voltammetry (CV) and galvanostatic charge-discharge cycling were used. CV was performed on a VMP3 potentiostat/galvanostat (Bio-Logic Science Instruments Co., Seyssinet-Pariset, France) with a scanning speed of 0.1 mVs^−1^ in the potential range of 0.01–2.0 V vs. Li/Li^+^ to study the main redox reactions that occur during lithiation and delithiation of the composite anode material. Galvanostatic cycling was carried out on a multichannel battery tester (Neware BTS-5V, Shenzhen, China and BT-2000 Arbin Instruments Inc., College Station, Texas, USA) in a range of potentials of 0.01–3.0 V vs. Li/Li+ and current density of 0.1 C (1 C = 2 mAh). All electrochemical measurements were carried out at room temperature. EIS measurements of uncycled SiNPs and Si-LTO-cPAN were carried out in the frequency range of 0.1 Hz–100 kHz with the amplitude of 5 mV s^−1^.

## 3. Results

**Figure 1:**
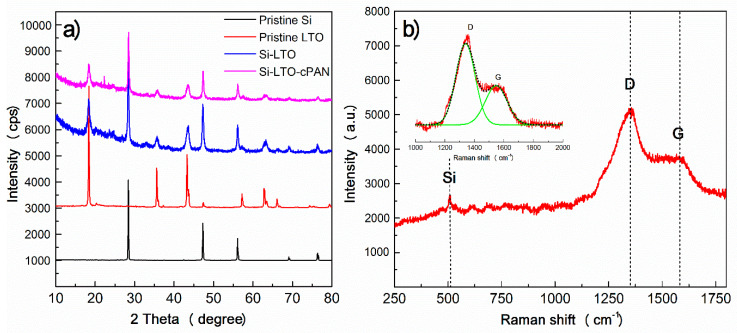
(**a**) X-ray diffraction (XRD) of pure Si nanoparticles (SiNPs), LTO powder, Si-LTO and Si-LTO-cPAN composites and (**b**) Raman spectrum of Si-LTO-cPAN composite.

**Figure 2:**
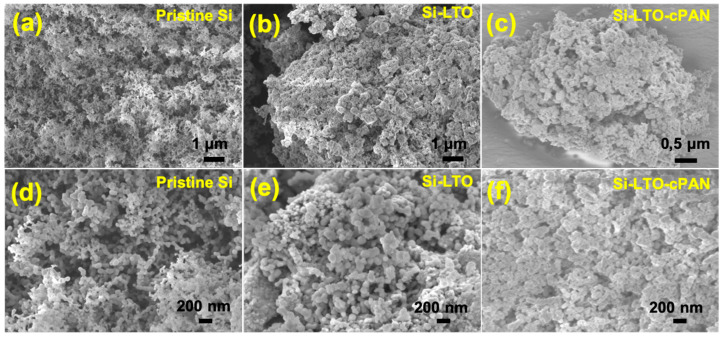
Scanning electron microscopy (SEM)pictures of pure SiNPs (**a**,**d**), Si-LTO composite (**b**,**e**) and Si-LTO-cPAN composite (**c**,**f**) at different magnifications.

All materials were characterized prior and after modification. Figure 1a presents the XRD spectra of the powders. As it can be seen, the pure Si powder shows several peaks that correspond to cubic crystalline form of Si according to JCPDS-ICDD-27-1402 [28]. The peaks corresponding to LTO in the Si-LTO and Si-LTO-c-PAN composite anodes indicate the formation of the cubic spinel structure after annealing in accordance with the JCPDS-49–0207. The characteristic peaks of anatase (TiO_2_) and rutile (TiO) at 25.26° and 27.39° can also be noticed, which can form impurities in small amounts [24]. Thus, all peaks of the composites can be indexed as characteristic peaks of pure components, which indicates that SiNPs retained their original crystalline structure in the composite after heat treatment and the spinel structure of LTO was formed upon annealing. From the Raman spectrum of Si-LTO-cPAN composite pictured in Figure 2b, we can observe the presence of delocalized sp^2^π D and G bands which corroborate the cyclization of PAN coatings [26].

Figure 2 shows the images of pure SiNPs along with Si-LTO and Si-LTO-cPAN composites. As can be seen in Figure 2a, SiNPs consist of spherical particles with a diameter less than 70 nm (Figure 2a,d). After coating with LTO, we can confirm from Figure 2b,c that the initial spherical morphology of Si was preserved. However, after heat-treatment with PAN (Figure 2c,g), although the particles almost retained their shape there are a slight increase in diameter and some agglomeration can be observed.

**Figure 3:**
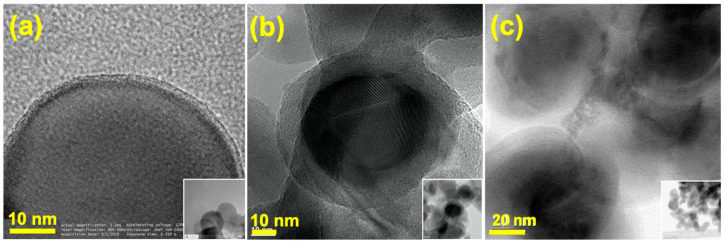
Transmission electron microscopy (TEM) pictures SiNPs (**a**), Si-LTO composite (**b**) and Si-LTO-cPAN composite (**c**) with corresponding insets.

TEM pictures of pure SiNPs (Figure 3a, inset) indicates individual particle sizes of up to 70 nm, which is consistent with the SEM results. It can be observed that silicon is coated with a very thin layer of silica (SiO_2_) of several nm, which is formed as a result of oxidation of the Si surface by oxygen of air [25]. In an enlarged image of Si in Figure 3a, atomic layers define the distance which corresponds to the Si reflection (111). After coating with LTO (Figure 3b), the thickness of the outer layer changed significantly increasing up to approximately to 10 nm. Here as well the Si crystal structure reflection can be noticed. The TEM image of the Si-LTO-cPAN composite in Figure 3c shows that along with the LTO the particle was enlarged by the presence of another layer, most likely carbonized PAN.

**Figure 4:**
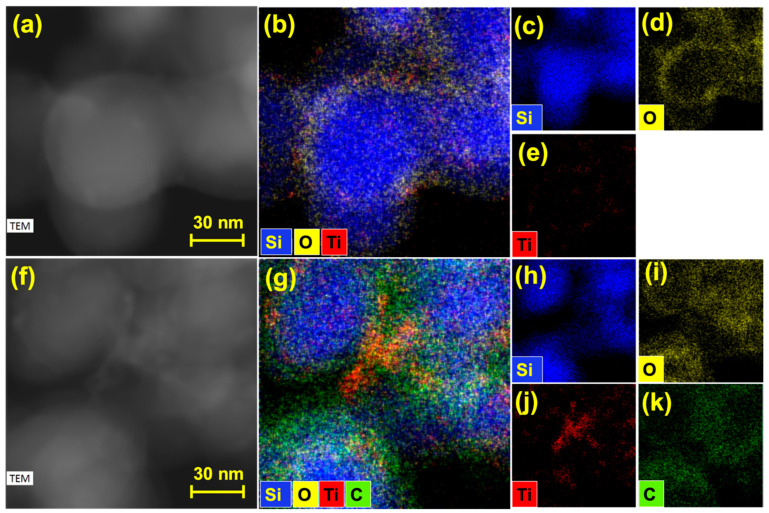
TEM and corresponding TEM-EDS pictures Si-LTO composite (**a**–**e**) and Si-LTO-cPAN composite (**f**–**k**).

To prove that each SiNPs were coated simultaneously with both materials, TEM-EDS images of the Si-LTO and Si-LTO-cPAN composites were provided in Figure 4. Figure 4a–d depicts the formation of a thin LTO layer on the SiNPs, which is indicated by the presence of O and Ti elements. The intensity of the O element is much higher considering that the O appears in both LTO and SiO_2_. After coating with cPAN, a conformal thin coating of approximately 5 nm of cyclized-PAN (green) can be observed on the surface of SiNPs. Along with the mechanical integrity, the coating makes a closely linked conductive network around SNPs which in turn link intimately the nanoparticles throughout the composite.

**Figure 5:**
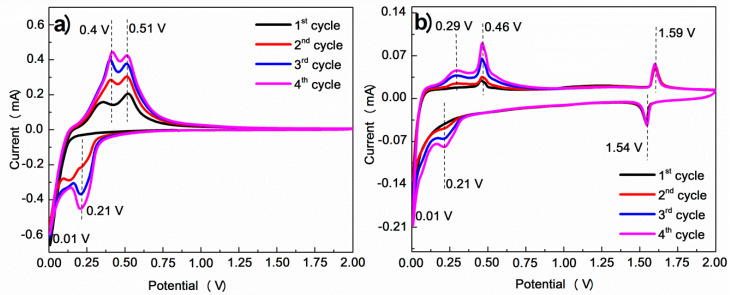
Cyclic voltammograms of (**a**) pristine SiNPs and (**b**) Si-LTO-cPAN composite at scan rate of 0.1 mV s^−1^ between 0.01–2.0 V.

Figure 5 shows the CV curves of the initial four cycles of the SiNPs and Si-LTO-cPAN composite. On the cathodic scan of the Si-LTO-cPAN composite, the peak at 1.59 V reflects the intercalation of the Li ion into LTO, which corresponds to a reversible phase transition between the spinel form LTO and the form of rock salt Li_7_Ti_5_O_12_. A sharp cathode peak at 0.21 V and below in both graphs corresponds to the working potential of Si and can be attributed to the alloying process of Si and Li with the formation of Li_x_Si. In the anodic scan direction, two typical peaks are present as the result of the decomposition of amorphous Li_x_Si into less lithiated alloys and/or Si. However, in the Si-LTO-cPAN composite the peaks are exhibited at a lower anodic potential compared with pristine SiNPs, inferring a smaller polarization and better electrochemical activity of Si-LTO-cPAN with enhanced charge transportation properties. Therefore, it can be concluded that the LTO layer enhanced the electronic conductivity and Li^+^ ion diffusivity of the SiNPs during charge/discharge cycling. Typically, due to the oxidation state of Ti, LTO is considered as a bad electronic conductor. Nonetheless, during lithiation process LTO changes to Li_7_Ti_5_O_12_ and Li_8.5_Ti_5_O_12_ which are good electronic conductors [29]. The anodic peak at 1.59 V corresponds to the deintercalation of Li^+^ ions from LTO, demonstrating the reversibility of intercalation of Li into the LTO matrix and the ability of LTO and Si to react with Li independently from each other. It is common to observe the activation phenomenon of Si that strengthens with cycling. As expected, cPAN stays inactive in the given range of potentials.

**Figure 6:**
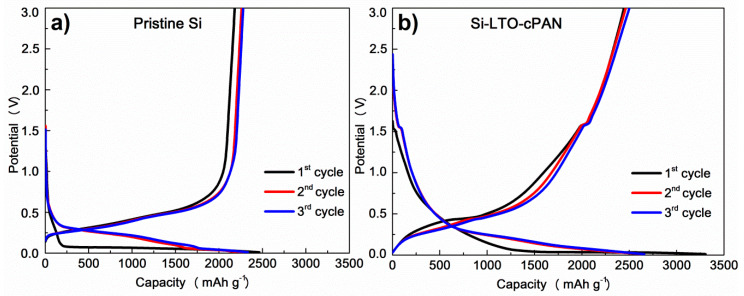
Galvanostatic charge-discharge of (**a**) pristine Si nanoparticles and (**b**) Si-LTO-cPAN composite.

Galvanostatic charge–discharge profiles obtained at a current density of 0.1 C to demonstrate the electrochemical properties are presented in Figure 6. All plateaus present in the curves perfectly coincide with the redox peaks CV voltammograms, indicating a highly reversible electrochemical process. Small plateaus belonging to lithiation and delithiation of LTO are present in the profile of modified SiNPs at around 1.5 V in Figure 6b. Initial specific capacities of 2181 and 2443 mAh g^−1^ were obtained for pristine SiNPs and Si-LTO-cPAN composite anodes, respectively. These capacities can be matched well to the removal of 2.7 mole of Li per mole of Si. Also, it is worth mentioning that the capacity of Si-LTO-cPAN composite anode was higher than that the previously reported cases with modified SNPs [24,25].

**Figure 7:**
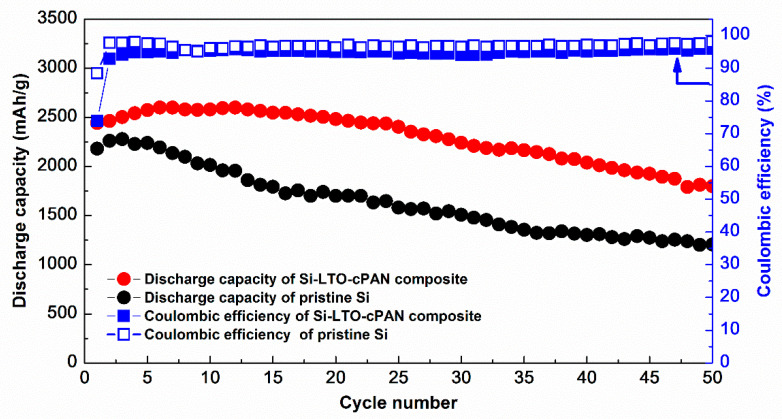
Capacity retention of pristine Si nanoparticles and Si-LTO-cPAN composite.

**Figure 8:**
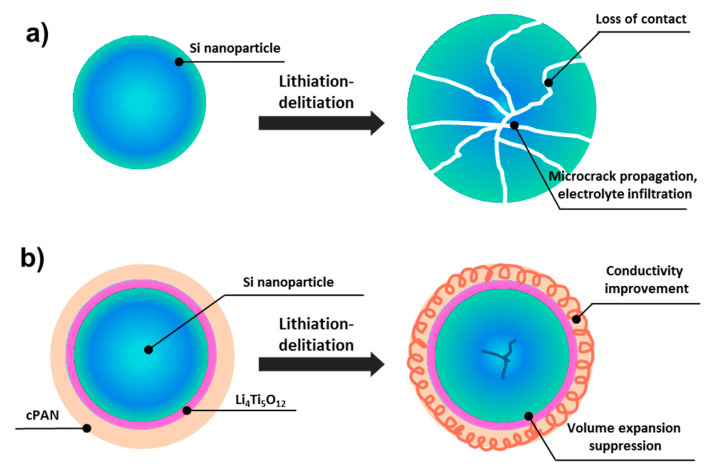
Schematic representations of failure mechanism of (**a**) pristine silicon nanoparticle and (**b**) improvements by LTO-cPAN coatings during cycling.

Capacity retention of Si-LTO-cPAN composite anode is shown in Figure 7. Observed initial increase in capacity can be explained by the activation (amorphization) of crystalline SiNPs. After 50 cycles the composite anode was able to retain 74% of its initial capacity with the Coulombic efficiency of 95%. The superior electrochemical performance of Si-LTO-cPAN composite anode is attributed to the unique morphology with fast charge transfer properties and reduced volume change during lithiation and delithiation processes. Figure 8 schematically explains the reason for the enhanced electrochemical stability of the Si-LTO-cPAN composite. It is expected that the pristine SiNPs go through particle deformation and cracking due to the volume expansion issues during cycling processes, while coating with LTO and PAN helps to preserve the morphology and mitigate the volume expansion. Moreover, conductive properties of PAN allow fast charge transfer in interconnected SiNPs network that stimulates cycling stability and higher specific capacity.

**Figure 9:**
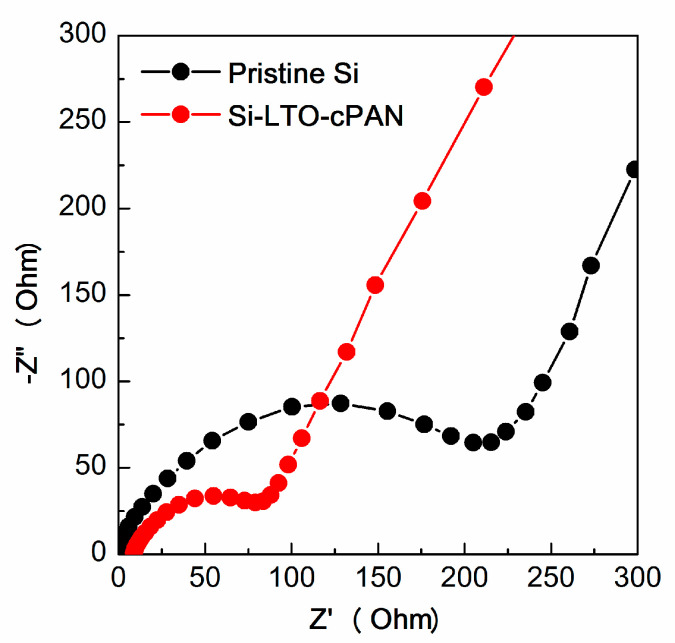
Nyquist plots of pristine Si nanoparticles and Si-LTO-cPAN composite.

Figure 9 illustrates the Nyquist plots of pristine and modified SNPs. Both samples possess one semicircle in high-medium frequency regions which is usually attributed to the charge transfer resistance and inclined line at low frequency region which is characteristic of Li^+^ ion diffusion in the solid. From the figure it can be clearly observed that the charge transfer resistance of the coated SNPs is much smaller in comparison to the pristine meaning that the two coated layers not only suppress the volume change but also helps to increase the conductivity.

## 4. Conclusions

In summary, we demonstrated a simple route to prepare the onion-like morphology SiNPs designed with the LTO and cPAN coatings, which delivers a reversible capacity of 2443 mAh g^−1^ at 0.1 C and long-term cycling stability with a capacity retention of 95% over 50 cycles. LTO and cyclized PAN coatings address several chronic issues which restrained the usage of SiNPs as an anode material. The onion-structure was designed to target the huge volume expansion and improve the conductivity: LTO being the zero-strain material can reduce the volume change while elastic polymer can help to reduce the volume change as well as to provide good conductivity.
